# Outcomes of drug provocation tests in children with chronic complications and comorbidities: Case series

**DOI:** 10.1016/j.jacig.2025.100436

**Published:** 2025-02-03

**Authors:** Kouhei Hagino, Kiwako Yamamoto-Hanada, Seiko Hirai, Daisuke Harama, Marei Omori, Yasuaki Matsumoto, Daichi Suzuki, Kotaro Umezawa, Fumi Ishikawa, Kenji Toyokuni, Tatsuki Fukuie, Yukihiro Ohya

**Affiliations:** a Allergy Center, National Center for Child Health and Development, Tokyo, Japan; b Department of Occupational and Environmental Health, Graduate School of Medical Sciences, Nagoya City University, Aichi, Japan; c Division of General Allergy, Bantane Hospital, Fujita Health University, Aichi, Japan

**Keywords:** Drug allergy, drug provocation test, chronic disease, children, delabeling

## Abstract

A study demonstrated that drug provocation tests could be conducted in children with underlying diseases, enabling accurate diagnosis of drug allergy. The study investigators hope that their work will allow more children with underlying conditions to undergo drug provocation tests.

Overdiagnosis of drug allergies in children remains a significant medical concern. Misdiagnosing a drug allergy can severely limit future treatment options once a child has been labeled as having a drug allergy. A cross-sectional study reported that only 4.4% of children with suspected drug allergies received an appropriate diagnosis following evaluation.^1^ The most frequently implicated medications were antibiotics, nonsteroidal anti-inflammatory drugs, and antipyretic analgesics.^1^ Another study similarly found that only 9.1% of children with suspected drug allergies were accurately diagnosed.^2^

Children with chronic complications and comorbidities are often prescribed multiple medications, making an accurate diagnosis of drug allergies crucial for appropriate treatment. Given the inherent risks of allergic reactions, including anaphylaxis, drug provocation tests (DPTs) must be conducted more cautiously in these children than in those without underlying conditions. It is not uncommon for such high-risk children to avoid undergoing DPTs owing to safety concerns. Furthermore, there is a scarcity of reports on the application of DPTs in children with chronic complications and comorbidities.^3^^,^^4^ This study aims to evaluate the efficacy and safety of DPTs in children with underlying diseases.

We conducted a retrospective cohort study of children admitted to the National Center for Child Health and Development for undergoing DPTs from January 2019 and December 2023. We collected electronic medical records and analyzed them in January 2024. The study protocol was approved by the ethics committee at the National Center for Child Health and Development (2023-2024). Informed consent was obtained in the form of an opt-out on the website. Eligibility criteria included being younger than 18 years and experiencing chronic complications and comorbidities requiring regular outpatient visits for other than allergic diseases. Suspected drugs were classified on the basis of medical history as immediate-type if symptoms appeared within 1 hour and as non–immediate-type if symptoms appeared after 1 hour.^5^ All children underwent skin prick tests (SPTs) for the suspected drug, and if the result was negative, intradermal tests (IDTs) were performed for injectable formulations. We conducted SPTs using optimal concentrations of the suspected drug, with histamine and control solutions as comparators.^6^^,^^7^ A positive result was defined as a wheal size at least half the diameter of the histamine wheal after 15 minutes.^8^ For IDTs, the suspected drug was prepared at an optimal concentration, and 0.02 mL was injected intradermally, with normal saline serving as the control solution. A positive result was defined as the appearance of a wheal with a diameter larger than 9 mm or an erythema with a diameter larger than 20 mm after 15 minutes.^9^ For children suspected of having non–immediate-type reactions, drug lymphocyte stimulation tests (DLSTs) were conducted. If the DLST results were 180% of the baseline or higher and the medical history strongly suggested positivity, a DPT was not performed. We conducted DPTs for drugs that showed negative results in all tests (SPTs, IDTs, or DLSTs). Leukotriene receptor antagonists and systemic antihistamines were discontinued 3 days before the SPTs, IDTs, and DPTs. DPTs were performed using various administration methods tailored to the individual disease type and history. For immediate-type reactions, children were stratified into low-risk and high-risk groups, with careful administration starting from a lower dose if necessary.

In this study, we included 14 children with chronic complications and comorbidities. Table I presents the characteristics of these children. Their median age was 12 years (range 2-18 years); the study sample included 7 males. Of the 14 children studied, 7 had a suspected allergy to a single drug and 7 were suspected of allergy to multiple drugs. The most common suspected drug category was β-lactam antibiotics, affecting 9 children. Immediate-type symptoms were observed in 8 children, non–immediate-type symptoms occurred in 5, and 1 case was unclassifiable. Two children had a history of anaphylaxis in response to suspected drugs.

**Table I:** Characteristics of the 14 children with chronic diseases and comorbidities who were receiving DPTs

Characteristic	Value
Age (y), median (min-max)	12 (2-18)
Male sex, no. (%)	7 (50)
Suspected drug type (yes), no. (%)	
Antibiotics (β-lactam)	9 (64)
Antibiotics (other)	2 (14)
NSAIDs (yes)	0
Local anesthetics (yes)	2 (14)
Contrast agent (yes)	2 (14)
Others (yes)	0
Timing of index reaction (yes), no. (%)	
Immediate reaction	8 (57)
Non-immediate reaction	5 (36)
Indeterminate	1 (7)
Anaphylaxis (yes), no. (%)	2 (14)
Doctor-diagnosed allergic diseases (yes), no. (%)	8 (57)
Presence of comorbidity (yes), no. (%)	14 (100)
Use of regular medication (yes), no. (%)	9 (64)
Immunosuppressive drug (oral)	6 (43)
Steroid (oral) (yes)	2 (14)
Others (yes)	8 (57)

*min-max,* Minimum-maximum; *NSAID,* nonsteroidal anti-inflammatory drug.

Table E1 (available in the Online Repository at www.jaci-global.org) presents the detailed results of the individual cases. The children had various chronic diseases and comorbidities, such as such as post–liver transplantation complications (n = 4), chronic respiratory diseases (n = 3), brain tumors (n = 2), and acute lymphoblastic leukemia (n = 1). In all, 3 case patients developed allergic reactions for suspected medications in the DPTs, with symptoms including erythema, urticaria, and itching, all of which were mild. No additional treatment was required during the DPTs, and we performed the DPTs safely.

To our knowledge, this report is the first case series evaluating the outcomes of DPTs in children with chronic complications and comorbidities. For children with chronic complications and comorbidities, it is crucial to avoid unnecessarily avoiding effective medications through accurate diagnosis. It is common that making a diagnosis in a child labeled as having a drug allergy restricts the use of first-choice medicines, leading to the use of less effective or more side effect–prone drugs. Furthermore, limited treatment options can increase medical costs. This study showed that DPTs could be conducted even in children with underlying diseases by adjusting loading dose and administration intervals according to individual cases.

Accurate diagnosis of drug allergies requires skin testing and DPTs.^10^ Recently, some protocols have recommended direct challenge tests without skin tests based on risk stratification. However, evaluating risk before performing DPTs in children with underlying diseases is challenging because unexpected events may occur. Therefore, at our hospital, we perform skin tests in all cases, and DLSTs for children suspected of non–immediate-type reactions. In cases in which obtaining sufficient blood samples is difficult owing to young age or underlying diseases, tests may not be performed. For these reasons, we conduct DPTs cautiously during hospitalization. Particularly for children using mechanical ventilation or having a history of anaphylaxis, we discuss how to take immediate actions if allergic reactions occur and perform DPTs in collaboration with the primary physician treating the underlying disease and pediatric intensive care unit faculties. Additionally, the DPT dose has started to be even lower than usual. We did not include children with heart failure in this study, and DPTs are generally contraindicated in such cases. We need to have more data about these children with heart failure.

Our study has a few limitations. First, the sample size was small, and we could not evaluate the follow-up status for all cases; however, our sample size represents the largest number of patients in a case series to date. We need more comprehensive data in the future. We are planning to conduct a prospective study and will be able to assess follow-up status in that future study. Second, we did not follow up with all the children after the DPTs.

## Disclosure statement

Supported by a grant from the 10.13039/100007786National Center for Child Health and Development.

Disclosure of potential conflict of interest: The authors declare that they have no relevant conflicts of interest.

## Supplementary data

Supplementary Table E1
